# Supplementary material for: Otogenic Meningitis or Meningoencephalitis in 30 Dogs: Association Between Neurological Signs, Magnetic Resonance Imaging Findings, and Outcome
Source: Vet Sci. 2025 May 9;12(5):456. doi: 10.3390/vetsci12050456 (PMC12115475; doi:10.3390/vetsci12050456)
Supplement: Supplementary file 1 [file vetsci-12-00456-s001.zip › vetsci-3560689-supplementary.pdf]

Table S1. Relationship between clinicopathological data and MRI classification in 30 dogs with otitis media-interna.* Group A: bulla/bullae occupation and changes in surrounding soft tissues without evidence of extension to the adjacent meninges or neuroparenchyma, Group B: bulla/bullae occupation, changes in surrounding soft tissues and imaging of meningeal post-contrast enhancement, Group C: bulla/bullae occupation, changes in surrounding tissues, meningeal thickening and/or enhancement and brainstem lesion with or without formation of an empyema. ME: Male entire, MS: Male spayed, FS: female spayed, CVS: Central vestibular syndrome, PVS: Peripheral vestibular syndrome, GCC: glucocorticoids, TNCC: Total Nucleated Count Cell, CSF: Cerebrospinal fluid.

	**MRI Group***			
**Parameter**	**A=6**	**B=17**	**C=7**	**Total number**
Age (years)	6	7	9.7	7.5 (mean)
Weight (Kg)	12.7	12.5	16.4	14.6 (mean)
Sex	ME 2<br>MS 2<br>FS 2	ME 4<br>MS 8<br>FS 5	ME 1<br>MS 1<br>FS 5	ME 7<br>MS 11<br>FS 12
Previous history of chronic or recurrent otitis externa	2	5	7	14
Otitis externa on admission	1	5	4	10
Use of antibiotic before diagnosis	2	6	3	11
Use of NSAIDs before diagnosis	1	2	2	5
Use of GCC before diagnosis	2	5	2	9
Clinical course	Acute 4<br>Chronic 2	Subacute 8<br>Acute 5<br>Chronic 4	Subacute 1<br>Acute 1<br>Chronic 5	Subacute 9<br>Acute 10<br>Chronic 11
Grade of vestibular ataxia	Mild 4<br>Moderate 2	Mild 11<br>Moderate 4<br>Severe 2	Mild 1<br>Moderate 2<br>Severe 4	Mild 16<br>Moderate 8<br>Severe 6
Neuroanatomical localization on admission	CVS 1<br>PVS 5	CVS 4<br>PVS 12<br>Multifocal 1	CVS 2<br>PVS 2<br>Multifocal 3	CVS 7<br>PVS 19<br>Multifocal 4
CSF TNCC median (cells/µl)	216	2748	9395	4707 (mean)
CSF Proteins median (mg/dl)	48	70	188	96.5 (mean)
NSAIDs treatment	3	7	1	11
Prednisolone treatment	3	10	4	17
Follow-up MRI	0	3	2	5
Follow-up CSF	0	2	1	3
Persistent neurological deficits	2	14	4	20
Euthanasia	0	0	2	2

Table S2. Ordinary Least Squares (OLS) regression results for MRI and selected variables in 30 dogs with otitis media-interna.

**Variable**	(1) **Intracranial extension in MRI**	(2) **Intracranial extension in MRI**	(3) **Intracranial extension in MRI**
Intracranial neurolocalization	0.300***	0.307**	0.424
	(0.106)	(0.122)	(0.274)
		-0.005	-0.097
Subacute clinical signs (baseline: acute)		(0.206)	(0.355)
		-0.033	-0.023
Chronic clinical signs (baseline: acute)		(0.210)	(0.227)
Moderate ataxia (baseline: mild)			-0.114
			(0.271)
Severe ataxia (baseline: mild)			0.106
			(0.292)
NSAIDS (previous treatment)			-0,338
			(0.315)
GCC (previous treatment)			-0,116
			(0.198)
Antibiotics (previous treatment)			-0,054
			(0.169)
Chronic or recurrent otitis			0,095
			(0.218)
External otitis on admission			-0,154
			(0.234)
Leucocytosis in blood			0,18
			(0.170)
R-squared	0.125	0.126	0.224
N	30	30	30

Notes: OLS regressions with robust standard errors in parentheses, *** denotes significance at the 1% level, ** 5% level and * 10% level.

Table S3. Ordinary Least Squares (OLS) regression results for the presence of sequels and selected variables in 30 dogs with otitis media-interna.

**Variable**	(1)<br>**Sequels**	(2)<br>**Sequels**
Intracranial extension in MRI	-0.485**	-0.671
	(0.217)	(0.537)
External otitis on admission		-1.112***
		(0.276)
Chronic or recurrent otitis		0,038
		(0.287)
Enrofloxacine		0.371
		(0.258)
Trimethoprim		0.580
		(0.460)
Metronidazole		-0.681
		(0.645)
Marbofloxacine		-0.758**
		(0.341)
Amoxiciline		0.272
		(0.499)
Cefalexine		0.510
		(0.488)
NSAIDS (previous treatment)		-0,189
		(0.431)
GCC (previous treatment)		-0,445
		(0.498)
Antibiotics (previous treatment)		-0.782**
		(0.367)
Treatment (weeks)		0.075
		(0.057)
GCC treatment (days)		0.003
		(0.003)
R-squared	0.194	0.962
N	28	17

Notes: OLS regressions with robust standard errors in parentheses, *** denotes significance at the 1% level, ** 5% level and * 10% level.
